# Synthesis and Microwave Absorbing Properties of Porous One-Dimensional Nickel Sulfide Nanostructures

**DOI:** 10.3389/fchem.2018.00405

**Published:** 2018-10-11

**Authors:** Min Lu, Qian Wu, Xiao-Hui Guan, Wei Xu, Hao-Yue Zhang, Xin Di, Guang-Sheng Wang, Shao-Hua Dong

**Affiliations:** ^1^School of Chemical Engineering, Northeast Electric Power University, Jilin, China; ^2^School of Chemistry, Beihang University, Beijing, China; ^3^Pipeline Technology Research Center, China University of Petroleum–Beijing, Beijing, China

**Keywords:** porous, nickel sulfide, one-dimensional, dielectric loss, microwave absorption

## Abstract

One-dimensional (1D) porous Ni_x_S_y_ nanostructures have been successfully fabricated by two-step method consisting of solvothermal and subsequent annealing process. The suitable heat treatment temperature and reaction time play crucial roles in the final structure, morphology, as well as performance. The uniform and perfect porous Ni_x_S_y_ nanostructures obtained at 310°C exhibit outstanding microwave absorption performances. A minimum reflection loss of −35.6 dB is achieved at 8.5 GHz, and the effective absorption bandwidth almost covers 14.5 GHz with the absorber thickness range of 2.0–5.0 mm. It can be supposed that this porous structure with rough surface which is favor for increasing the microwave multiple reflection and scattering, contributes a high-performance electromagnetic absorption.

## Introduction

Microwave absorber with strong capacity in absorption, low proportion in filler loading, thin thickness in coating, and wide bandwidth in absorption frequency, has aroused burgeoning research interest because of their great potential applications both in military and civil fields, including stealth technology, information security, electromagnetic interference shielding, and healthcare (Zhu et al., 2010; Zhao H. et al., 2014). As is well known that many factors such as morphology, geometry and structure, have vital impacts on determining the microwave absorption (MA) properties (He et al., 2013). Conventional microwave absorbers with different morphologies that have been divided into three classes are as follows: (1) one-dimensional nanostructures such as ZnO nanowires (Wang et al., 2014), Bi_3_S_2_ nanorods (Luo et al., 2014); (2) two-dimensional materials such as MoS_2_ nanosheets (Ning et al., 2015), α-Fe_2_O_3_ flakes (Lv et al., 2015a); and (3) three-dimensional network structures, including Co_20_Ni_80_ hierarchical nanospheres (Liu et al., 2015), Ni chains nets (Liu et al., 2016), Fe_3_O_4_@carbon ordered arrays (Yuan et al., 2015) and so on. However, the aforementioned materials usually possess high density, thus leading to severe limitations to their practical applications in some specialized fields. In this regard, materials with characteristics of low density and special void spaces, such as yolk–shell structural microspheres (Liu et al., 2013; Yu et al., 2014; Qiang et al., 2016), foam composites (Zhang Y. et al., 2015; Zhao H. B. et al., 2016), as well as porous nanostructures (Yan et al., 2009; Zhou et al., 2010; Zhu et al., 2011), are highly beneficial to obtaining superior microwave absorption performance.

Considerable attention has been concentrated on the porous structure of MA materials owing to its fascinating characteristics. For example, Liu et al. fabricated porous carbon/Co composites, and the results suggested that the composites with large dielectric loss could achieve a minimum RL of −40 dB at 4.2 GHz with a coating thickness of 5 mm (Liu et al., 2008). Lv et al. synthesized Co/CoO porous 3-D flower nanostructure through annealing process at 400°C and found that the minimal reflection loss was up to −50 dB when the coating thickness was 3.5 mm (Lv et al., 2015b). Similarly, Wang et al. reported the formation of porous flower-like NiO decorated graphene, and the composites with a filler loading of 25 wt% exhibited highly MA performance (−59.6 dB) because of their special porous structures and numerous void spaces (Wang et al., 2017). Benefiting from the porous structure, the above-mentioned materials show superior electromagnetic wave absorption performance, which reveals that these porous materials are effective as MA materials.

Metal sulfides, as semiconductor materials, have been proven to have promising potential as an ideal microwave absorber on account of their typical dielectric loss mechanism (Zhang X. J. et al., 2017). Recent studies have suggested that metal sulfides including MoS_2_ (Wang et al., 2015), CoS_2_ (Zhang C. et al., 2017), CdS (Zhang et al., 2014), and CuS (He et al., 2014), as well as various phase of nickel sulfides (Zhou et al., 2010) can effectively absorb electromagnetic waves and attenuate them in the form of thermal energy. However, the microwave-absorbing properties of Ni_x_S_y_ with special porous structure have not been reported previously. Based on the above study, we demonstrated the successful design and fabrication of porous one-dimensional Ni_x_S_y_ nanomaterial through a facile solvothermal route together with annealing process. The MA properties of resultant composites were investigated in detail for the first time. As expected, the synthesized Ni_x_S_y_ nanostructure exhibited excellent microwave absorption property confirming that this material can be used as high-performance microwave absorber.

## Materials and methods

### Preparation of porous Ni_x_S_y_

Nickel nitrate hexahydrate (Ni(NO_3_)_2_·6H_2_O), elemental sulfur, ethylene glycol (EG), and ethylenediamine (EN) were purchased from Nanjing Chemical Reagent Co. All of the chemical reagents were analytical-grade purity and used without further purification.

Typically, nickel sulfide was synthesized by the reaction of Ni(NO_3_)_2_·6H_2_O, EN and sulfur powder in EG. At first, Ni(NO_3_)_2_·6H_2_O (0.3489 g) was added to EG (135 mL) under strong magnetic stirring to form a light green homogeneous solution. Then the sulfur powder (0.0288 g) was dissolved in EN (15 mL) through ultrasonic treatment. Mix the two solutions together and put it into oil bath, maintained at 120°C for 6 h. After being cooled to room temperature, the resulting solid precursors were centrifuged, washed with alcohol to remove possible remnant, and finally dried in air at 60°C for 24 h. The dried precursors were treated at 310°C for 2 h with a heating rate of 2°C/min under N_2_ atmosphere to get the final Ni_x_S_y_ products.

### Preparation of nickel sulfide/PVDF nanocomposites

The polyvinylidene fluoride (PVDF) was first dispersed in *N-N* dimethylformamide (20 mL) under magnetic stirring for 1 h. Then, the desired amount of nickel sulfide was added into the suspension. After ultrasonication for another 1 h, the mixture was poured onto a glass plate and dried at 80°C for 24 h. The samples for testing were also compacted into a cylindrical compact (Φ_*out*_ = 7.00 mm and Φ_*in*_ = 3.04 mm) by hot pressing at 210°C under 5 MPa (pressed for 15 min, followed by cooling to room temperature under the same pressure).

### Instrumental analyses

The X-ray diffraction (XRD) pattern of the nickel sulfide product was carried out on a Rigaku, Dmax2200 diffractometer equipped with a CuKa radiation source (λ = 1.5416 Å) in the range of 2θ = 10–80°. For the phase analysis. Further microstructural analyses were performed by using a FEI Quanta 250 field emission gun environmental scanning electron microscope (JSM-6700F microscope) at 15 kV. In brief, SEM samples were prepared by diluting the final products with alcohol by ultrasonic treatment and dropping it on the silicon slice. The relative permittivity (*ε*′, *ε*″) and permeability (*μ*′, *μ*″) values were measured using two-port vector network analyzer (Agilent E5071C) over the frequency of 2–18 GHz at room temperature, coupled with a coaxial wire setup. Finally, the reflection loss (RL, dB) value which presents the ratio of the total reflected microwave power against the incident microwave power can be calculated by using the following formulas (Abbas et al., 2006; Xu et al., 2018).

(1) $$\begin{array}{rcl} Z_{in} & = & \sqrt{\frac{\mu_{r}}{\varepsilon_{r}}}\tanh\left[j\left(\frac{2f\pi d}{c}\right)\sqrt{\mu_{r}\varepsilon_{r}}\right] \end{array}$$

(2) $$\begin{array}{rcl} RL\left(dB\right) & = & 20\log|\frac{Z_{in}-1}{Z_{in}+1}|\; \end{array}$$

where Z_in_ is the normalized input characteristic impedance, *f* is the frequency of microwave, *d* is the thickness of the absorber. A lower RL value stands for a better MA performance.

## Results and disscussion

An illustration of the synthesis of the Ni_x_S_y_ is shown in Figure S1. We first prepared the precursors of Ni_x_S_y_ nanorods by a simple solvothermal method in a controlled way as described later. Then the collected dried precursors were transferred into a tube furnace and annealed at 310°C for 2 h under an N_2_ atmosphere, which eventually led to the generation of the porous Ni_x_S_y_. It is well recognized that reaction parameters such as temperature, pressure, reaction time, type of solvent, and concentration of reagents, have a huge effect on the morphology of the products. In the present reaction system, Ethylene glycol and Ethylenediamine were applied as solvents for the precursors synthesis of Ni_x_S_y_, and the temperature as well as reaction time was tightly regulated so that the precursors at a well-defined state were obtained.

**Figure 1:**
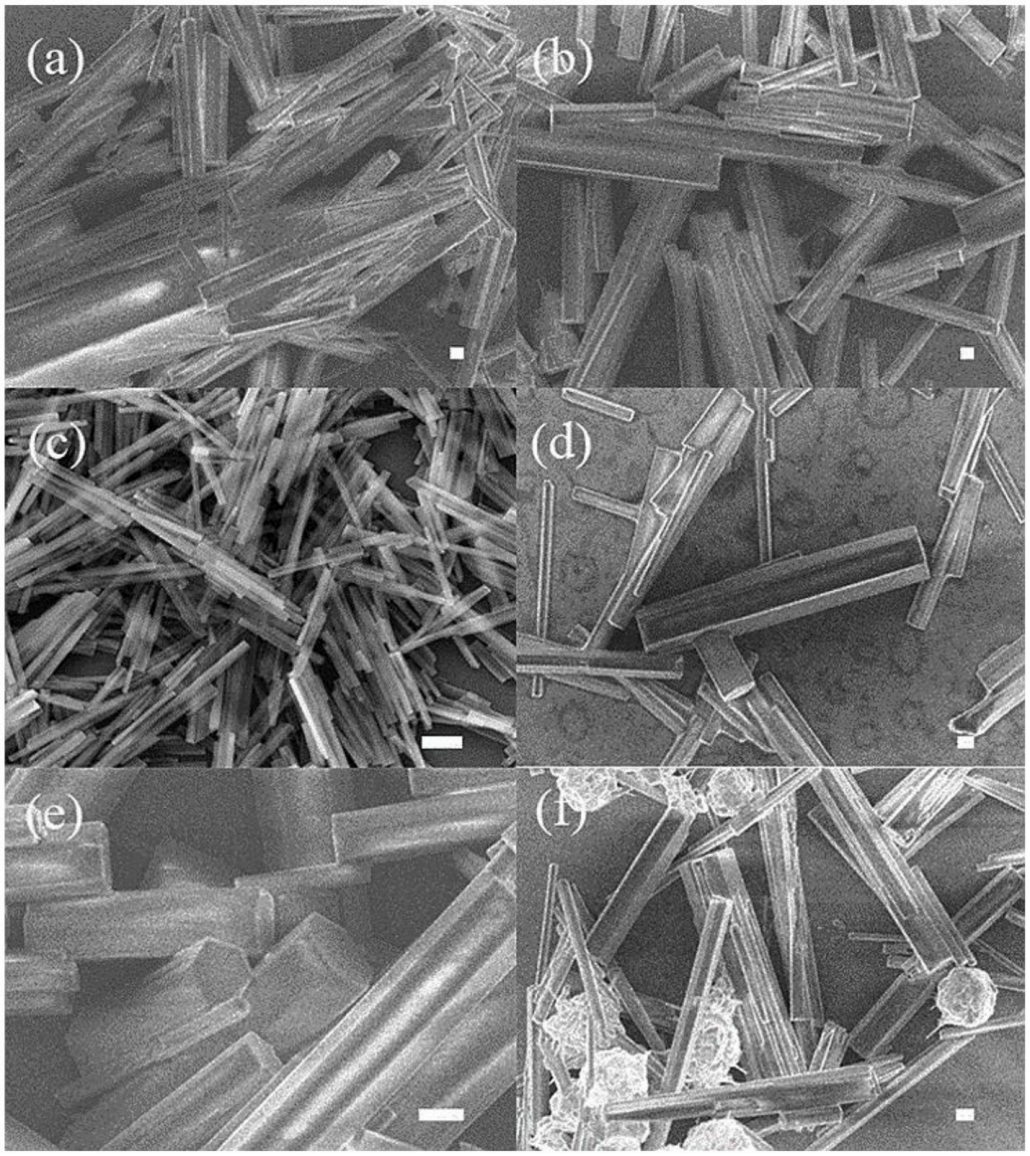
SEM images of precursor of Ni_x_S_y_ samples under different reaction temperatures: **(a)** 80, **(b)** 100, **(c)** 120, **(d)** 140, **(e)** 160, and **(f)** 180°C. (scale bar = 1 μm).

Figure 1 shows the representative SEM images of the precursors prepared at different temperature of 80, 100, 120, 140, 160, and 180°C, respectively. When the reaction is performed under the lower temperature (80 and 100°C), although one-dimensional structures of products can be formed, their thicknesses and lengths are varying greatly. On the contrary, when the temperature reaches 180°C, the 1D nanostructures are nonuniform, and a certain number of spherical impurities emerge. By comparing the morphology of the products under different temperature conditions, the best reaction temperature is determined. The products are well-defined nanorods with diameters of about 50 nm and lengths of several micrometers at the suitable reaction temperature of 120°C (Figure 1c). On the other hand, the stages of the growth process are monitored at 120°C by varying the reaction time from 2 to 10 h (Figure S2). It is interesting to find that reaction time does not change the overall morphology of the precursors. However, the yield of the products is very low within a short period of 2 h, indicating the slow reaction rate in such reaction system. Further increasing the reaction time produce more products, while the diameter and structure of the nanorods remain stable.

**Figure 2:**
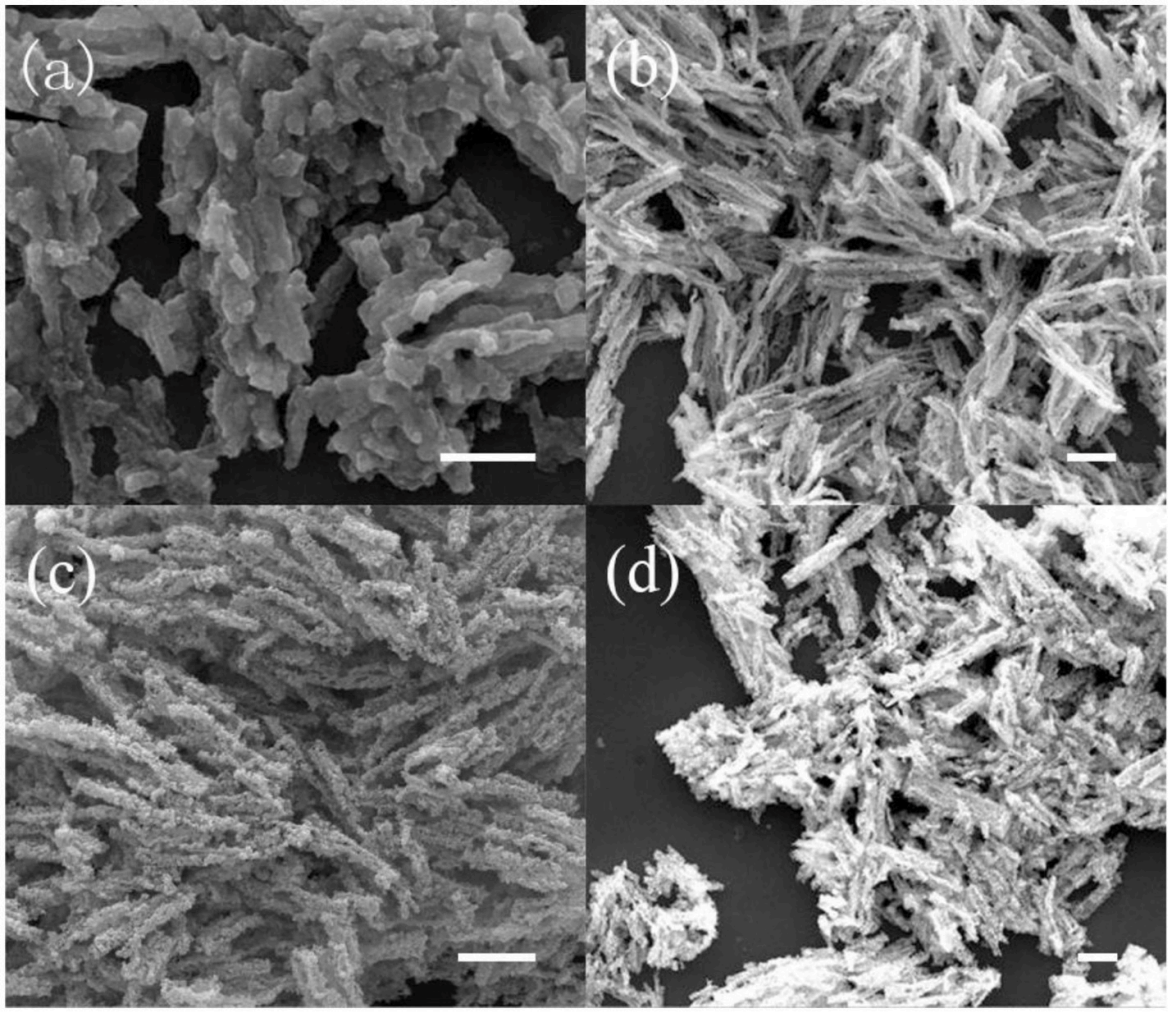
SEM images of Ni_x_S_y_ samples under different annealing temperatures: **(a)** 280, **(b)** 300, **(c)** 310, and **(d)** 330°C. (scale bar = 1 μm).

**Figure 3:**
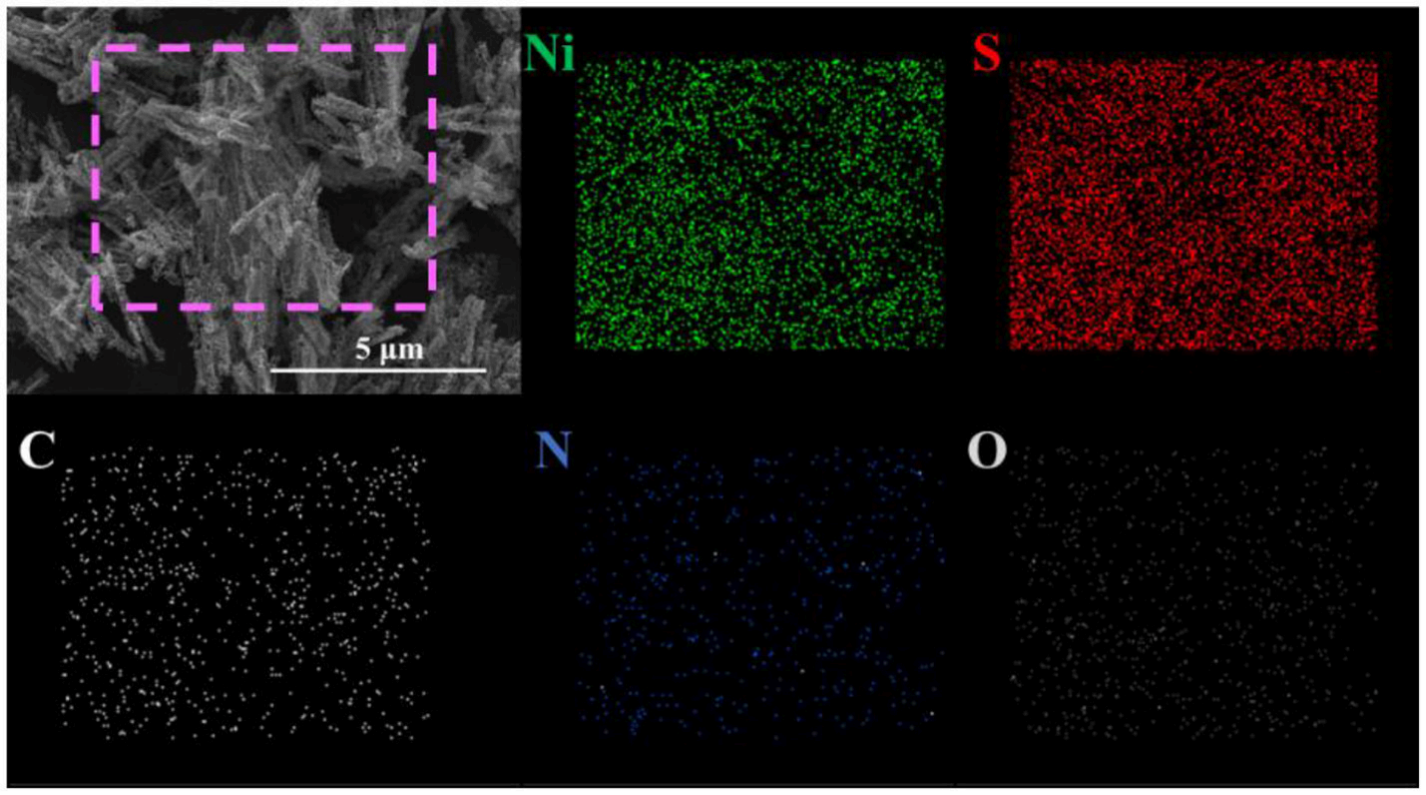
FESEM image of the Ni_x_S_y_ nanorods and corresponding elemental mapping images of Ni, S, C, N, and O.

To obtain porous 1D nanostructures, the precursors were annealed at different temperatures ranging from 280 to 330°C under the flowing nitrogen gas, and the final products are exhibited in Figure 2. It can be seen that the surfaces are actually becoming porous with annealing temperature, and the as-obtained Ni_x_S_y_ retain the rods morphology with appropriate porosity when prepared at 310°C. Further increasing the annealing temperature will led to 1D structural instability and collapse. The energy-dispersive X-ray spectroscopy (EDS) indicates that the obtained product is composed of Ni, S, C, N, and O elements, also demonstrating a very homogeneous elemental distribution (Figure 3). It is worth mentioning that the C, N, and O element signals originate from the incomplete decomposition of organic compositions of precursors during heat treatment. Meanwhile, the XRD results suggest that the as-synthesized products are poorly crystallized, containing NiS_2_ phase and Ni_7_S_6_ phase (Figure S3).

**Figure 4:**
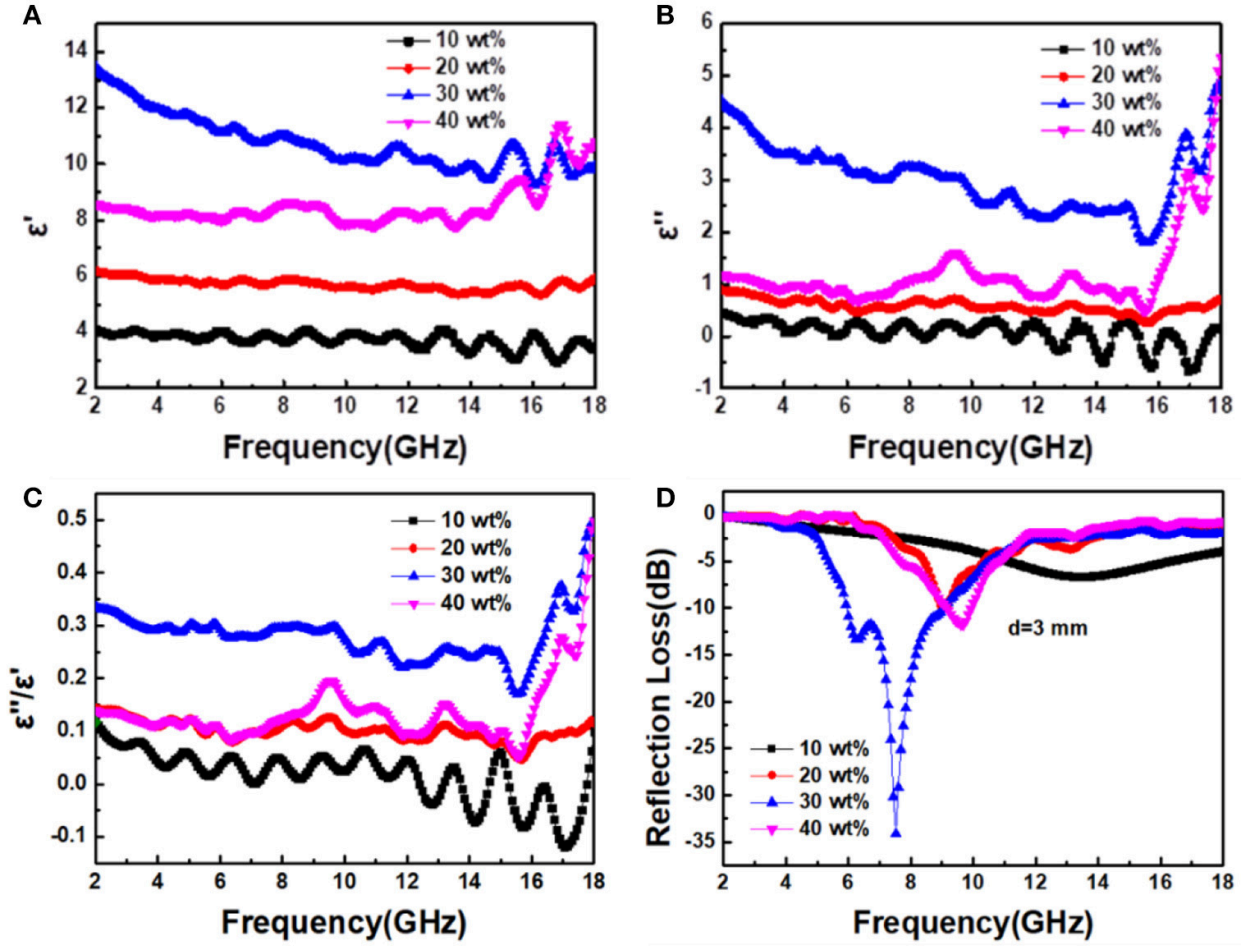
Frequency dependence of **(A)** real part and **(B)** imaginary part of permittivity, **(C)** dielectric loss tangent, and **(D)** reflection loss under a 3 mm thickness for NixSy composites with different filler loadings.

Figures 4A,B show the frequency dependence of the real part (*ε*′) and imaginary part (*ε*″) of the complex permittivity for Ni_x_S_y_/PVDF composites with 10, 20, 30, and 40 wt% filler loadings in the frequency range of 2–18 GHz. Generally, the *ε*′ is known to stand the storage capability of electromagnetic energy, and *ε*″ associated with various of polarization present the energy dissipation (Zhang X. et al., 2015; Zhao B. et al., 2016). As can be seen in Figure 4, with the increase of Ni_x_S_y_, the *ε*′ and *ε*″ values show a similar tendency. For nanohybrids with low concentration of filler (10 and 20 wt%), the values of *ε*′ and *ε*″ are approximately equal to some certain constant in the whole frequency range (*ε*′ = 4, *ε*″ = 0.5, and *ε*′ = 6, *ε*″ = 1). With the Ni_x_S_y_ proportion increasing from 20 to 30 wt%, the *ε*′ increases from 6 to 13 and the *ε*″ changes from 1 to 4.5 at 2 GHz. However, when the proportion of fillers is increased to 40 wt%, both the *ε*′ and *ε*″ dramatically decrease, which is possibly due to the fact that the higher concentration of Ni_x_S_y_ in this nanohybrid may result in severe agglomeration. Similar phenomena could be observed in dielectric loss tangent (tan δ_*e*_ = *ε*″/*ε*′) which is universally applied to evaluate the dielectric loss capacity of the microwave absorber (Yang et al., 2017), shown in Figure 4C. It can be seen that the dielectric loss tangent increases with the Ni_x_S_y_ proportion first, getting a maximum value of 0.35 with 30 wt% Ni_x_S_y_, and then decreases to a value of 0.1.

**Figure 5:**
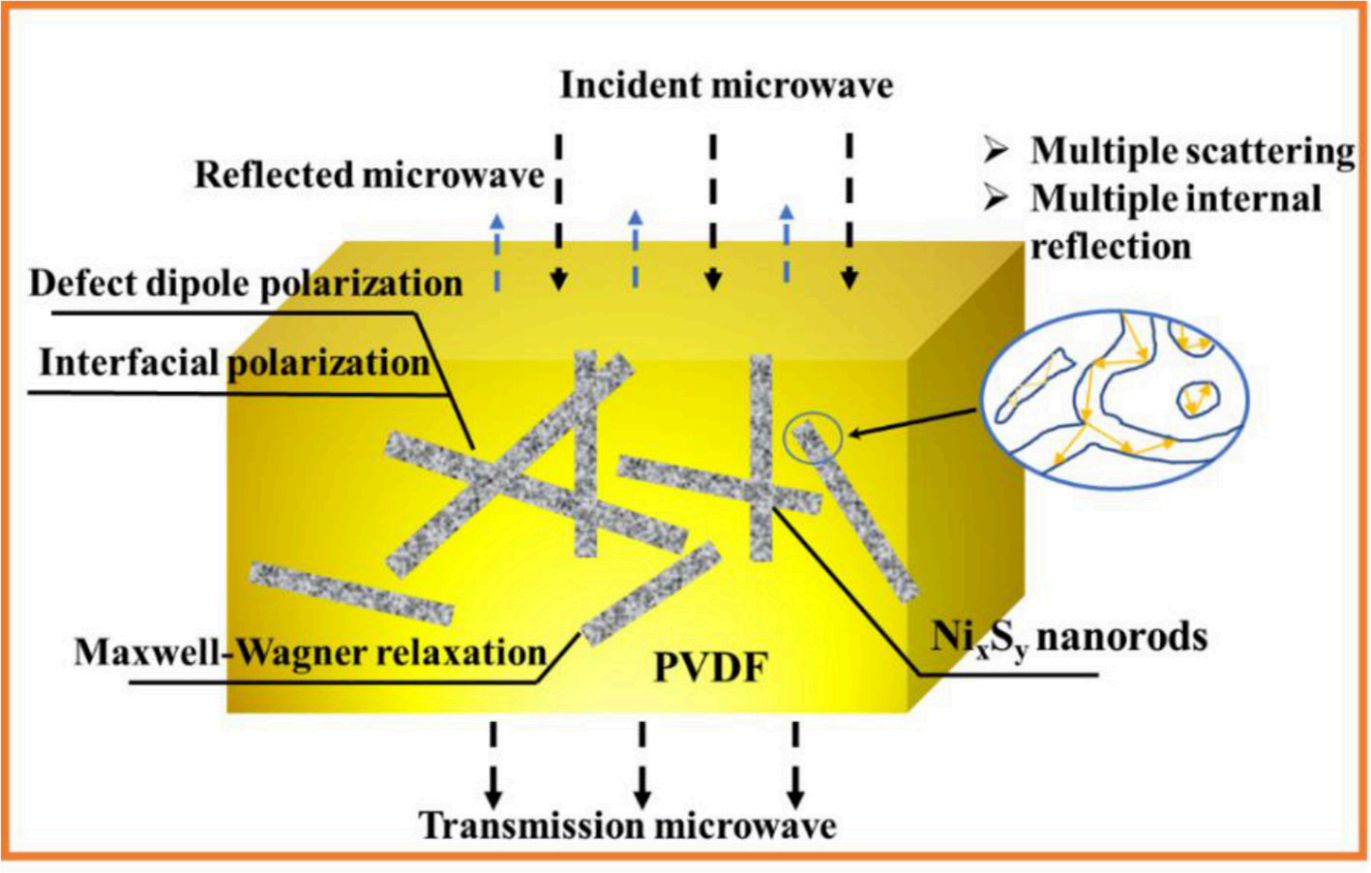
Possible mechanisms of microwave absorption for porous Ni_x_S_y_/PVDF composites.

On the basis of the above analysis, it can be deduced that the nanohybrids with 30 wt% Ni_x_S_y_ possess the best microwave absorption properties which is highly consistent with the test results, shown in Figure 4D. The minimum RL reaches −34 dB at 7.5 GHz with a thickness of 3 mm, indicating that 99.9% of incident electromagnetic wave is attenuated. Since Ni_x_S_y_ is a typical semiconductive material, various polarization, and related relaxation resulting in a strong dielectric loss are the dominant mechanism for microwave attenuation (Zhao et al., 2015). The dielectric loss of Ni_x_S_y_ mainly originate from the defect dipole polarization, the interfacial polarization, and the electronic relaxation loss. First, the defect dipoles are generated by the charge unbalance around the sulfur vacancies in the Ni_x_S_y_ lattice, while the interfacial polarizations come from the existence of plentiful interfaces between porous fillers and polymer matrix. Moreover, the porous structures of fillers with rough surfaces further induce the multiple reflection and scattering, resulting in more longer propagation path and greater energy loss (Figure 5). Second, the carbonation of precursors benefits electrical conduction, while the internal doping with nitrogen favors electron transport thus further improving electrical conduction. Furthermore, the calcination also induces a structural disorder and defects into NixSy that frequently lead to an enhanced electrical activity. This calcination process results in significant electronic relaxation polarization, which would enhance the dielectric loss obviously.

**Figure 6:**
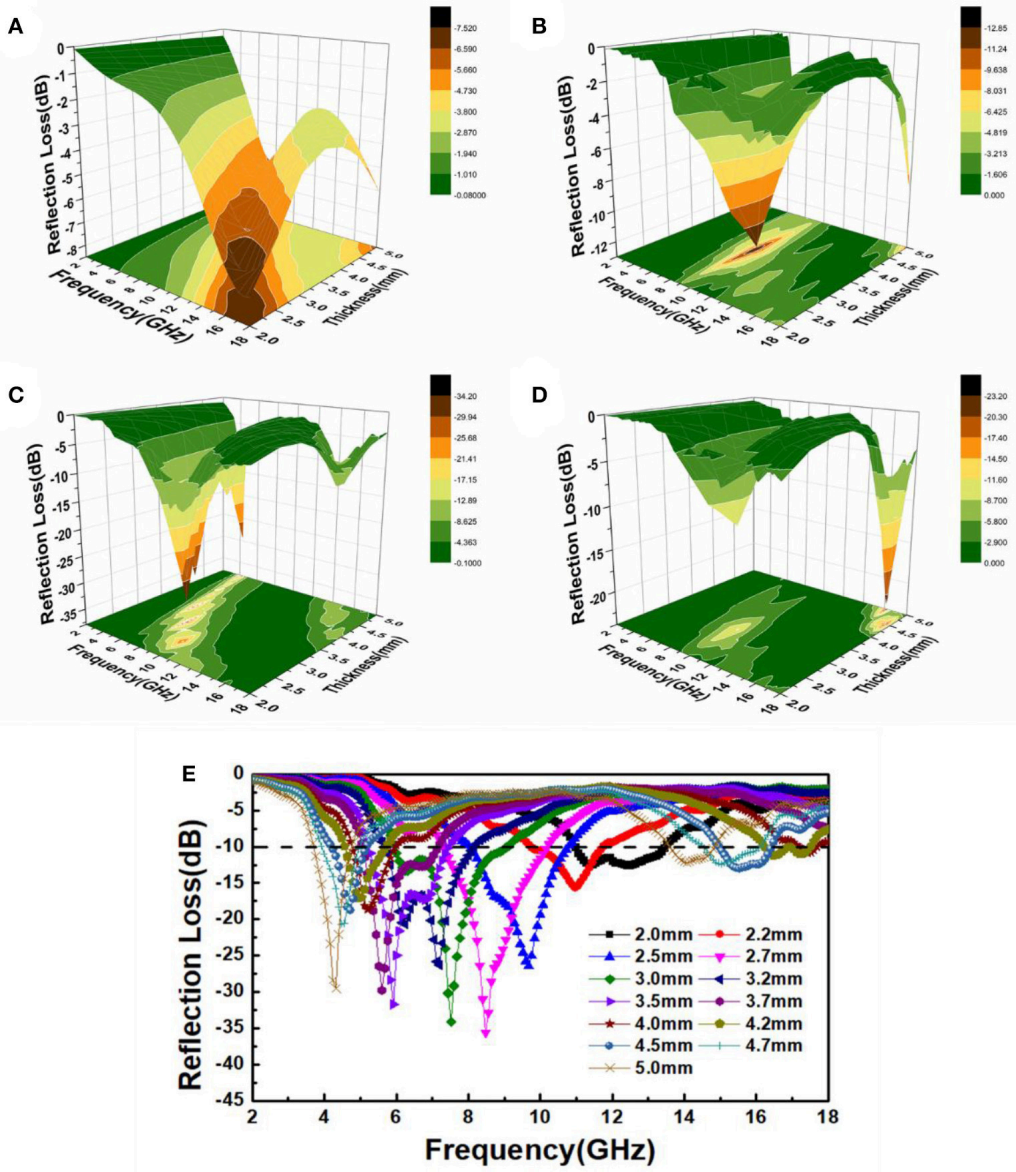
Three-dimensional presentations of the reflection loss for the Ni_x_S_y_ composites with different filler loadings: **(A)** 10, **(B)** 20, **(C)** 30, and **(D)** 40 wt%. **(E)** RL curves for 30 wt% NixSy composites with different thickness.

Figures 6A–D shows three-dimensional presentations of calculated reflection loss for the Ni_x_S_y_/PVDF with different filler loadings. Clearly, the composite with 30 wt% Ni_x_S_y_ has the best performance, and the RL values under different thickness are shown in Figure 6E. The minimal reflection loss of −35.6 dB is obtained at 8.5 GHz with a coating thickness of 2.7 mm and the effective bandwidth is about 3 GHz. Furthermore, the RLs exceeding −10 dB in the frequency range of 3.68–18 GHz are obtained for a variation in absorber thicknesses of 2.0–5.0 mm, demonstrating that this kind of materials has great potential for use as a microwave absorber. Meanwhile, there is an interesting phenomenon that with the increasing thickness of absorber the RL peaks shift to the lower-region frequency. This phenomenon is consistent with a so-called quarter-wavelength (λ/4) matching model (Deng and Han, 2007; Wang et al., 2011, 2013) which plays another significant role in electromagnetic attenuation. The model can be expressed as $t_{m}=n\lambda /4=nc/4f_{m}\sqrt{\left|\mu_{r}||\varepsilon_{r}\right|}\left(n=1,\;3,\;5\right)$, where |μ_*r*_| and |*ε*_*r*_| are the moduli of μ_*r*_ and *ε*_*r*_, respectively. Besides, when the thickness of absorber satisfies above equation, the curves corresponding to wavelengths of λ/4 (crescent shape) can be observed (seen in Figure 6C).

## Conclusions

In summary, we have successfully demonstrated an approach for the large-scale production of 1D porous Ni_x_S_y_ nanostructures via solvothermal synthesis together with an annealing process. Through controlling the reaction temperature and time, products with uniform morphology are obtained. The results reveal that the interesting porous structure of Ni_x_S_y_ might benefit the access of incident microwave and offer more active sites for multiple reflections and scattering, and thereby improve microwave absorbing performance. The minimum RL of −35.6 dB is achieved with a thickness of 2.7 mm at −35.6 dB GHz. The absorption bandwidth with RLs below −10 dB is up to 14.5 GHz when considering thicknesses of 2.0–5.0 mm. Combining the synergistic effect with intrinsic chemical properties and special structures, the Ni_x_S_y_ nanorods are promising for utilization as MA materials in various fields, such as aeroplanes and spacecraft.

## Author contributions

ML designed experiments, carried out experiments, analyzed experimental results, and wrote the manuscript. QW, WX, H-YZ, and XD helped experimental result. X-HG gave guidance on the revision of the experimental plan and manuscript. G-SW and S-HD funded experimental topics.

### Conflict of interest statement

The authors declare that the research was conducted in the absence of any commercial or financial relationships that could be construed as a potential conflict of interest.
