# Supplementary material for: Synthesis and Microwave Absorbing Properties of Porous One-Dimensional Nickel Sulfide Nanostructures
Source: Front Chem. 2018 Oct 11;6:405. doi: 10.3389/fchem.2018.00405 (PMC6193059; doi:10.3389/fchem.2018.00405)
Supplement: Supplementary file 1 [file Data_Sheet_1.docx]

**Supplementary Materials**

**Synthesis and Microwave Absorbing Properties of Porous One-Dimensional Nickel Sulfide Nanostructures**


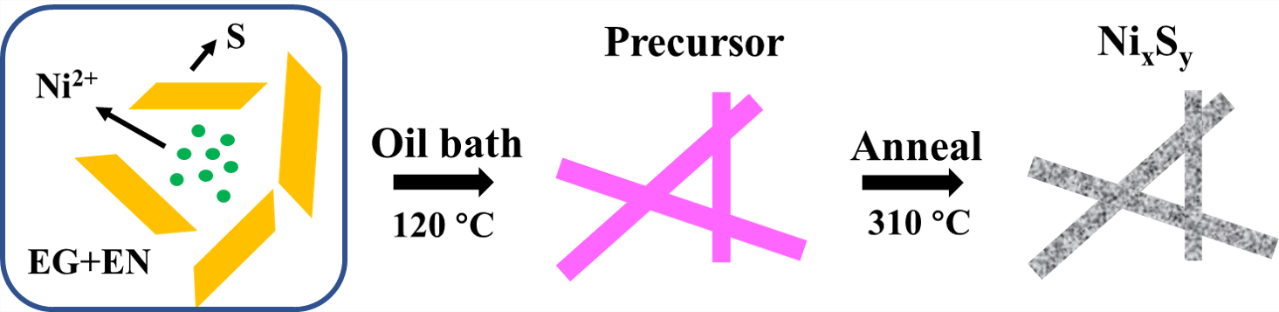


**Figure S1 | Growth mechanism of porous 1D nanorods.**


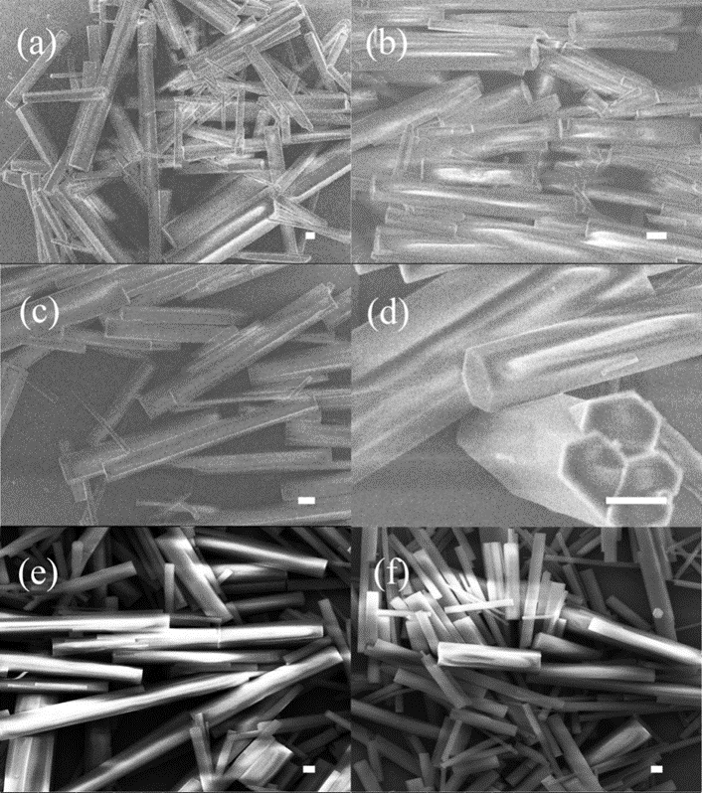


**Figure S2 | SEM images of precursor of Ni_x_S_y_ samples under different reaction times: (a) 2, (b) 4, (c) and (d) 6, (e) 8, (f)10 h.** (scale bar = 1 μm).


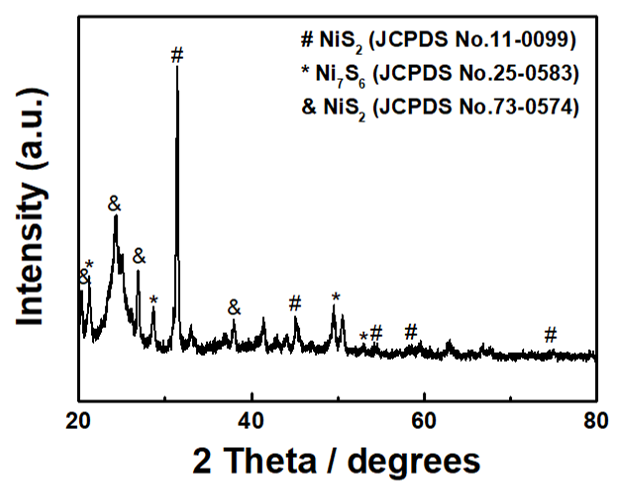


**Figure S3 | XRD pattern of as-synthesized Ni_x_S_y_ samples.**
